# *In vivo* sub-femtoliter resolution photoacoustic microscopy with higher frame rates

**DOI:** 10.1038/srep15421

**Published:** 2015-10-21

**Authors:** Szu-Yu Lee, Yu-Hung Lai, Kai-Chih Huang, Yu-Hsiang Cheng, Tzu-Fang Tseng, Chi-Kuang Sun

**Affiliations:** 1 Department of Electrical Engineering and Graduate Institute of Photonics and Optoelectronics, National Taiwan University, Taipei 10617, Taiwan; 2 Applied Physics Option, California Institute of Technology, Pasadena, CA 91125, USA; 3 Institute of Physics and Research Center for Applied Sciences, Academia Sinica, Taipei 115, Taiwan; 4 Molecular Imaging Center and Graduate Institute of Biomedical Electronics and Bioinformatics, National Taiwan University, Taipei 10617, Taiwan.

## Abstract

Microscopy based on non-fluorescent absorption dye staining is widely used in various fields of biomedicine for 400 years. Unlike its fluorescent counterpart, non-fluorescent absorption microscopy lacks proper methodologies to realize its *in vivo* applications with a sub-femtoliter 3D resolution. Regardless of the most advanced high-resolution photoacoustic microscopy, sub-femtoliter spatial resolution is still unattainable, and the imaging speed is relatively slow. In this paper, based on the two-photon photoacoustic mechanism, we demonstrated a *in vivo* label free laser-scanning photoacoustic imaging modality featuring high frame rates and sub-femtoliter 3D resolution simultaneously, which stands as a perfect solution to 3D high resolution non-fluorescent absorption microscopy. Furthermore, we first demonstrated *in vivo* label-free two-photon acoustic microscopy on the observation of non-fluorescent melanin distribution within mouse skin.

Microscopy based on non-fluorescent absorption dye staining, since its introduction 400 years ago, is currently widely used for various medical and biological researches. Unlike its fluorescent counterpart, non-fluorescent absorption-contrast microscopy lacks a methodology to provide *in vivo* imaging with a 3D sub-femtoliter resolution, thus limiting its applicability. It is primarily used under bright field microscopes. Photoacoustic microscopy (PAM), a promising branch of optical absorption 3D microscopy based on non-radiative emission, provides manifold morphologic and functional information in biomedical and molecular imaging applications, both *in vivo* and *ex vivo*^1^. In common PAM systems, implementation is usually based on an acoustic–optical confocal configuration. Either the optical excitation spot or the acoustic detection spot determines the lateral resolution, while the axial resolution comes from the impulse response of the ultrasonic detector used. Varying these engineering parameters sustains resolution scalability, which allows PAM to support a scalable imaging field of view (FOV) from macroscopic to microscopic domains^2^. Regardless of the capability of resolving non-fluorescent contrasts, current PAM is not able to provide the essential 3D sub-femtoliter resolution *in vivo*.

The lateral resolution in PAM is usually promoted by using objective lenses with a high numerical aperture (NA)^3^. In the most advanced PAM using a numerical aperture (NA) 1.23w objective lens, a high lateral resolution of 0.22 μm has been demonstrated *in vivo*^4^. On the other hand, the axial resolution is enhanced by using wide-bandwidth ultrasonic transducers^5^. The wider the bandwidth, the finer the resulting axial resolution. This is a further indication that signal detection needs to involve acoustic waves of higher frequencies. Recently, with a 100 MHz bandwidth transducer, a deconvolved 7.6 μm high axial resolution of PAM was reported *in vivo*^5^. Even though 1 μm axial resolution could be attained by further raising photoacoustic frequency, less than 10 μm penetration is an ineludible expense^6^. In this somewhat extreme case, the superiority of PAM in penetration depth compared to other optical microscopy has already been sacrificed. Moreover, despite the existence of the abovementioned submicron-level lateral and 10-micron-level axial resolution of PAM for *in vivo* imaging applications, a combination of these is hard to be realized. Sub-femtoliter-resolution non-fluorescent absorption 3D microscopy remains a distant prospect. Another issue with *in vivo* high-resolution PAM is the imaging rate. It is because that the FOV is limited by the small detection volume of the focused ultrasonic transducer and that pixel duration is restricted by the repetition rate of nanosecond lasers^7^. For instance, 125 MHz ultrasonic transducer only provides ~60 μm shift invariance^5^. Therefore, most current high-resolution PAM could only work with mechanical scanning stages. It usually takes tens of minutes to complete a single image, which is slow compared with other laser-scanning *in vivo* imaging modalities^4^,^8^.

In the past few years, several groups have discussed the two-photon acoustic contrast for microscopy and spectroscopy^9^,^10^,^11^. Due to the intrinsic localization of photoacoustic excitation provided by a non-radiative two-photon absorption (TPA) mechanism, it is hypothesized that both high lateral and high axial resolution could be attained simultaneously. Sub-femtoliter spatial resolution non-fluorescent imaging becomes possible. Also, because of the optically determined spatial resolution, there is no need to involve high frequency ultrasound. Large acoustic FOV makes the translation of ultrasonic transducer unnecessary, thereby allowing fast optical scanning. Furthermore, utilization of near infrared (NIR) femtosecond lasers at 800~1300 nm wavelengths for two-photon excitation is known to offer deep penetration^12^. The comparison between common PAM and two-photon acoustic microcopy (2PAM) is shown (Fig. 1). In spite of the advantages and previous investigations, *in-vivo* sub-femtoliter resolution 2PAM has not been reported yet. To separate the two-photon acoustic signal from its single-photon background, we recently proposed the use of a loss modulation technique^13^. In this previous study, the extraction of pure two-photon acoustic signals was realized by narrow-band electronic filtering and the two-photon nature was confirmed by power dependency tests.

In this paper, we report our realization of *in vivo* 2PAM with a sub-femtoliter spatial resolution. This was achieved with two-photon acoustic contrast excited by a Ti:sapphire femtosecond laser, combining a loss modulation system to remove the linear photoacoustic background. We optimized the efficiency of two-photon acoustic excitation by ameliorating a material dispersion effect, carefully characterized the spatial resolution of 2PAM in both lateral and axial directions, and solved the imaging speed problem by integration with a galvo-scanner. In resolution characterization, our study supported an optically determined spatial resolution in both axial and lateral directions. With a high NA objective, sub-femtoliter spatial resolution was thus achieved. Finally, we demonstrated the first *in vivo* laser-scanning high-frame-rate 2PAM based on non-fluorescent absorption contrasts with a sub-femtoliter 3D spatial resolution. For *in vivo* imaging applications, we find this 2PAM to be ideal for high spatial resolution imaging of melanin distribution in animal skin.

## Results

### Power dependency study with an exogenous two-photon acoustic contrast agent

**Figure 2:**
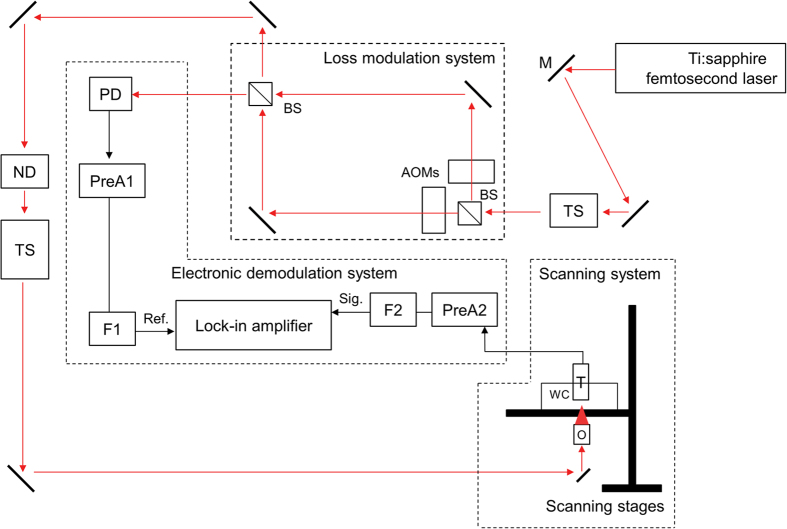
Experimental setup of 2PAM based on mechanical scanning. The red arrows in the figure represent laser light paths, while the black arrows indicate electrical signal connections. M, mirror; TS, telescope; BS, non-polarizing beam-splitter; AOM, acousto-optic modulator; PD, photo detector; ND, neutral density wheel; O, objective lens; WC, water cavity; T, ultrasonic transducer; PreA1 and PreA2, amplifiers (PR5678 and PR5660B, Olympus) for pre-amplification; F1 and F2, electronic low-pass filters (BLP-15+ and BLP-1.9+, Minicircuit); Ref., reference signal in the electrical form; Sig., photoacoustic signals in the electrical form.

**Figure 3:**
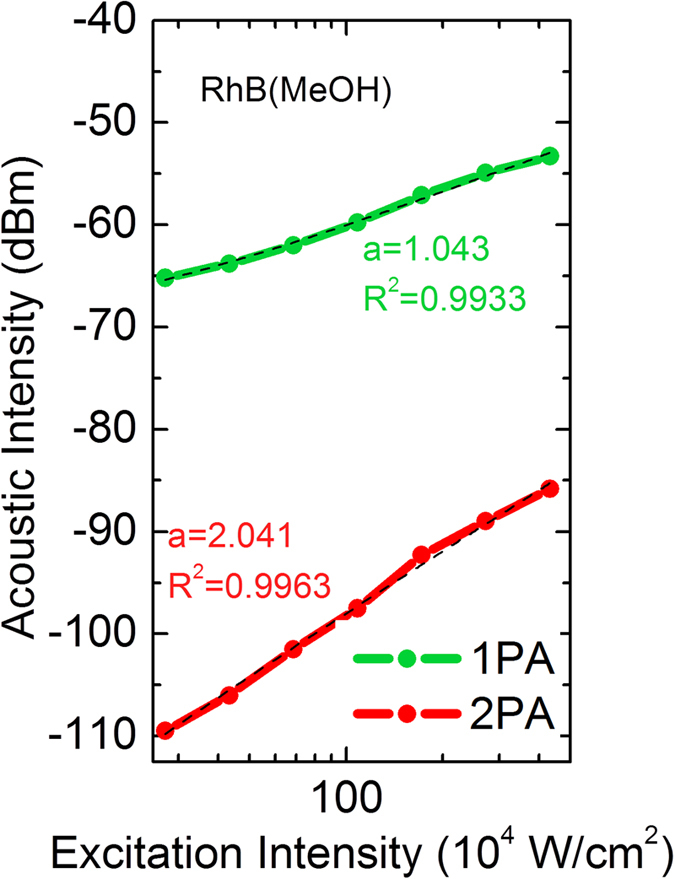
The power dependency of the photoacoustic signals within Rhodamine B solution (100 mM Rhodamine B in methanol, RhB(MeOH)). Reference lines (black dashed lines) with fitted slopes are given. a, best-fitted power dependence slope; R^2^, R-squared value of linear regression.

Here, we used Rhodamine B (Sigma Aldrich) in a methanol solvent as our two-photon acoustic contrast agent for the power dependency study, the investigation of material dispersion effect, and the resolution characterization due to its strong two-photon absorption characteristics at the NIR wavelength^14^. We first tested its power dependency to verify the two-photon nature of our detected signal. A Ti:sapphire laser (Spectra Physics Tsunami, *λ* = 800 nm, rep. rate = 80 MHz) with ~100 fs pulse width was used as the light source for 2PAM system^13^ (Fig. 2; *see also* Two-photon acoustic microscopy system setups *in* Methods). The laser was intensity modulated with a pure sinusoidal envelope and then focused onto the Rhodamine B solution (100 mM) with a NA0.3 objective lens (MPLFLN 10×, Olympus) for photoacoustic excitation. In this experiment, the modulation frequency, *f*, was 0.5 MHz. An immersion-type ultrasonic transducer (V303, Olympus) with a 1 MHz central frequency (ultrasonic focal spot was 2 × 2 × 9 mm^3^) was used for photoacoustic signal detection. A lock-in amplifier (LIA, SR844, Stanford Research) with a reference signal demodulated the photoacoustic signals at *f*, 0.5 MHz, referring to signals from single-photon absorption (1PA) or at 2*f*, 1 MHz, referring to signals from two-photon absorption (2PA) (Fig. 3). Reference lines with fitted slopes are provided in Fig. 3. Here, the preamplifier gave +60 dBm pre-amplification. The quadratic dependence of the 2PA (2*f*) signals indicates that we were truly collecting two-photon-absorption induced photoacoustic signals.

### Effect of material dispersion and dispersion compensation

**Figure 4:**
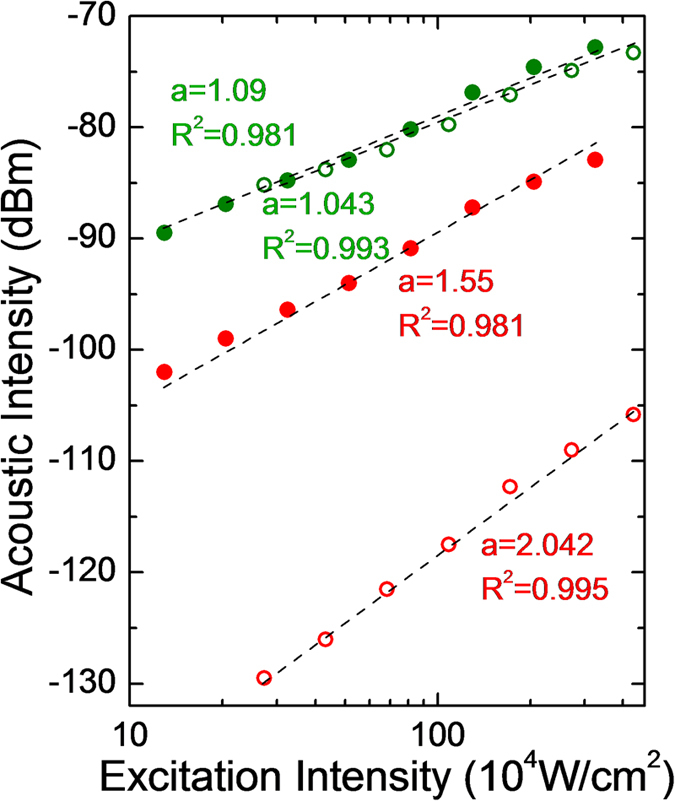
The green spots represent 1PA signals, demodulated at 0.5 MHz, and the red spots stand for 2PA signals, demodulated at 1 MHz. In addition, the hollow spots represent photoacoustic signals without compensation, and the solid spots indicate signals with compensation. Reference lines (black dashed lines) with fitted slopes are given. a, fitted power dependence slope; R^2^, R-squared value of linear regression.

Owing to the use of acousto-optic modulators in the loss modulation system, optical pulse broadening occurs due to material dispersion, which degrades the two-photon excitation efficiency^15^,^16^. Therefore, we inserted a prism pair (AFS-SF14, Thorlabs) after the loss modulation system to compensate the material induced dispersion^17^. A commercial auto-correlator (AC-100 ps, UVisIR) and a spectrometer (WaveScan) were used to measure the pulse width and the pulse bandwidth before and after the prism pair to see whether laser pulses had been compressed back. We found that, for a laser pulse with a pulse width ~100 fs, fine compensation was achieved when the prism pair was of a ~65 cm separation distance, indicating that the overall material positive dispersion was ~20000 fs^2^. The result was consistent with other literatures^18^,^19^. This serious positive dispersion caused the pulse to be broadened to ~560 fs, which might severely degrade the two-photon excitation efficiency. To verify this point, after the quantification of material dispersion, we compared the photoacoustic signals of the Rhodamine B solution with and without dispersion compensation. The modulation frequency, *f*, for excitation was 0.5 MHz; a NA0.3 objective lens (MPLFLN 10×, Olympus) was used for light focusing; LIA (SR844) was used for signal demodulation in this experiment (Fig. 2). The photoacoustic signals detected before and after the dispersion compensation are shown (Fig. 4), and reference lines with fitted slopes are also provided. Here, preamplifier gave +40 dBm pre-amplification. As we can see in the figure, dispersion compensated laser pulses were able to induce much stronger 2PA signals than uncompensated pulses. However, in the 1PA signals, intensities showed no dependence on the laser pulse width. This is because that the strength of the linear signals only depends linearly on the average excitation power and is not pulse-width dependent. The experimental results thus agreed with our expectation. Through the dispersion compensation, we significantly improved the signal to noise ratio of 2PAM system.

In Fig. 4, it is worth noting that the slope of 2PA signals with compensation appears to be smaller than the original quadratic dependence without compensation. It is because that although shorter optical pulses could excite stronger TPA effect and raise the induced acoustic intensity, stronger TPA also leads to easier TPA saturation^20^,^21^. From a literature^22^, we know that demodulated signals would show saturation and milder slopes at high optical excitation intensity with intense population transition within the used contrast. Therefore, we attribute the smaller slope of 2PA signals with compensation to TPA saturation. This indicates that in future applications, the selection of dye is important. The dye that can sustain strong TPA without saturation will be highly desirable for 2PAM application. For those dyes saturated under strong TPA, one would lower the optical excitation intensity to avoid TPA saturation. However, the induced acoustic intensity would inevitably become weaker, and the two issues should be compromised when deciding the optimal optical excitation intensity.

### Spatial resolution characterization of two-photon acoustic microscopy

From theory, 2PAM should possess high 3D spatial resolution microscopic imaging, which is the benefit from the excitation of photoacoustic waves based on nonlinear optical absorption. Via the intrinsic TPA excitation confinement, the lateral resolution 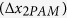 and axial resolution 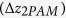 of 2PAM should be determined by the TPA point spread function as^19^,


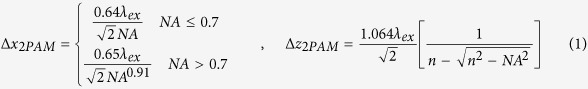


where *λ*_*ex*_ is the excitation wavelength, and *n* is the medium refractive index. The spatial resolution, especially the axial resolution, is independent of the frequency of the photoacoustic waves. Not only submicron lateral- and micron axial- resolution could be achieved at the same time, a lower-frequency ultrasound waves (around 1 MHz, rather than 50–100 MHz) could be used to allow deeper detection.

**Figure 5:**
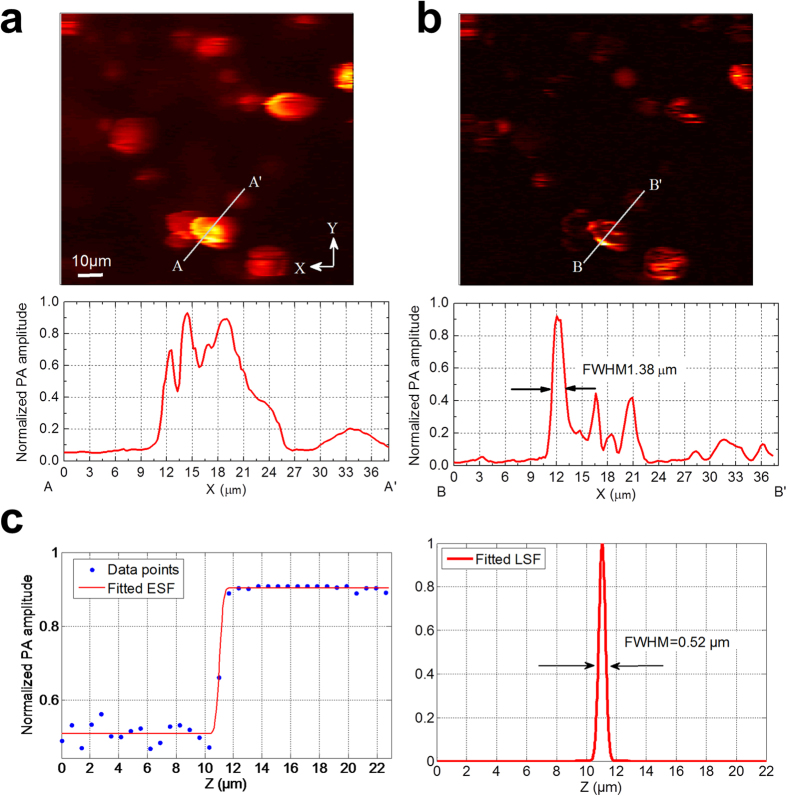
Mechanically scanned images of a Rhodamine B processed leaf sample with NA0.3 objective lens (MPLFLN 10×, Olympus) based on (**a**) demodulation at *f*, 0.5 MHz, (1PA) and (**b**) demodulation at 2*f*, 1 MHz, (2PA). The oval objects were verified as chloroplasts. From the plotted line profile, we derived the upper bound for lateral resolution of 2PAM, with this objective lens, to be 1.38 μm. (**c**) A polyethylene (PE) tube filled with Rhodamine B solution was imaged with a NA0.9w objective lens (LUMPLFLN 60×, Olympus) in the lateral direction. The interface between the PE tubule wall and the Rhodamine B solution provided the step-edged function. The ESF was well fitted and the derived LSF gave us the lateral resolution. With this objective lens, the lateral resolution of 2PAM was measured as 0.52 μm, and R^2^ value, the goodness of fit, was 0.991.

We verified the lateral resolution of 2PAM either by selecting a small object in a 2D scanned image as the upper bound of the resolution or by applying a step-edged method^8^ (*see* **Step-edged method for axial resolution characterization** *in* **Methods**). The 2D imaging was completed with mechanical scanning (Fig. 2). One example of the former method with a NA0.3 objective lens is shown (Fig. 5a,b). Images from both 1PA and 2PA signals are presented for comparison. In addition, one example of the latter method with a NA0.9w objective lens is also shown (Fig. 5c).

**Figure 6:**
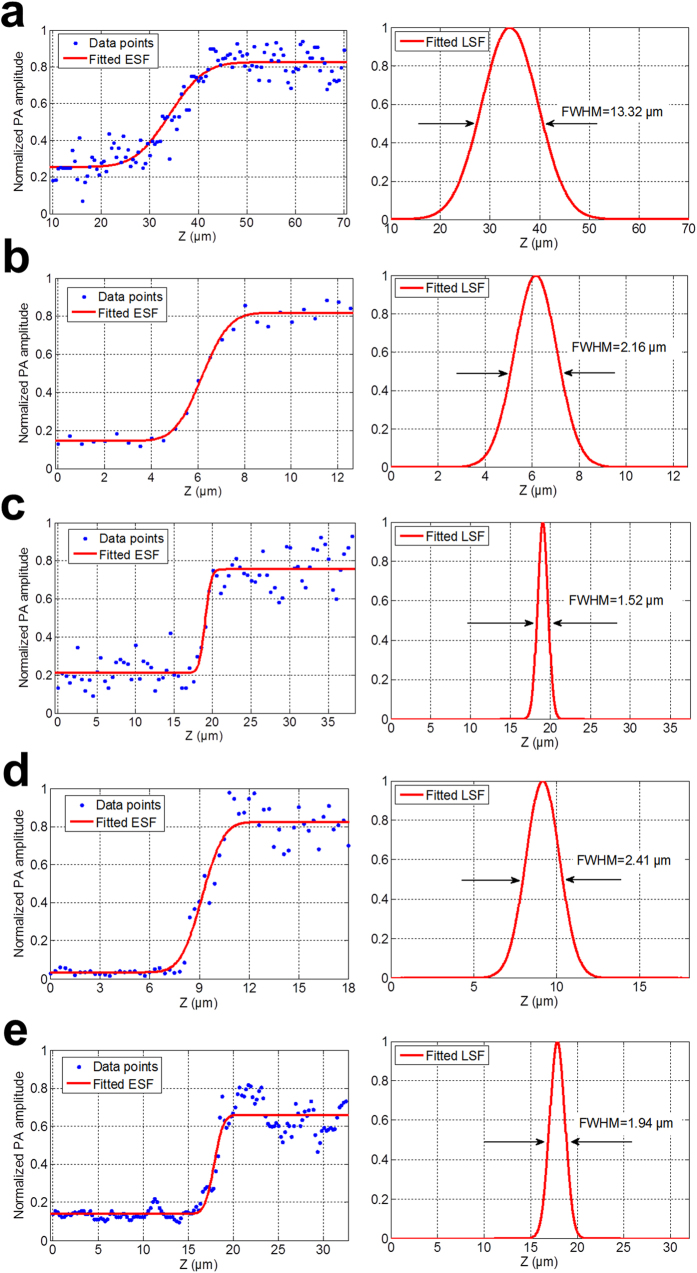
The interface between the coverslip and the Rhodamine B solution was imaged with several objective lenses with different NA in the axial direction. Both the fitted ESF and the derived LSF are shown in each condition including (**a**) NA0.3 objective lens (MPLFLN 10×, Olympus), R^2^ value, the goodness of fit, was 0.997 (**b**) NA0.7 objective lens (LCPlanFLN 50×, Olympus), R^2^ value was 0.989 (**c**) NA0.8 objective lens (MPlanFLN 50×, Olympus), R^2^ value was 0.915 (**d**) NA0.8w objective lens (LUMPLFLN 40×, Olympus), R^2^ value was 0.963, and (**e**) NA0.9w objective lens (LUMPLFLN 60×, Olympus) R^2^ value was 0.942.

On the other hand, we used the step-edged method to characterize the axial resolution of 2PAM. The interface between the coverslip and the Rhodamine B solution provided the step-edged function. We repeated the same procedure with several objective lenses of different NAs (Fig. 6). With a fixed ultrasound detection frequency, the axial resolution of 2PAM was heavily dependent on the NA of the objective lens used. The best experimental result, with a NA0.8 objective lens, was 1.52 μm. Since we used an ultrasonic transducer with a central frequency of only ~1 MHz, which would have corresponded to millimeter-poor resolving power if the resolution had been determined ultrasonically, this means that the fine axial resolution was resulted directly from the nonlinear absorption mechanism. Also, by using a NA0.9w objective lens, we achieved lateral and axial resolution upper bounds of 0.52 μm and 1.94 μm, respectively. The results support a sub-femtoliter ultra-high 3D spatial resolution.

**Figure 7:**
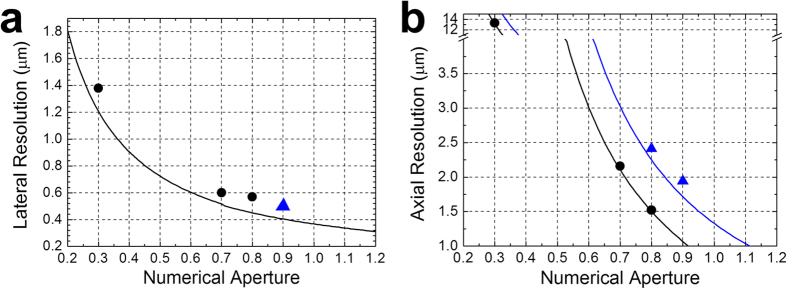
(**a**) The lateral and (**b**) axial resolutions of 2PAM from experimental and theoretical results with objective lenses of different NA. The solid (black/blue) {lines/dots} correspond to the {theoretical/measured} resolution with (air/water) objective lenses.

The lateral and the axial resolution of 2PAM is summarized and compared to that calculated from Eq. (1) (Fig. 7). We found that experimental results agreed well with the theoretical two-photon fluorescence resolution, thereby corroborating the expected high spatial resolution of 2PAM.

### Laser scanning two-photon acoustic microscopy by integration with a galvo-scanner

**Figure 8:**
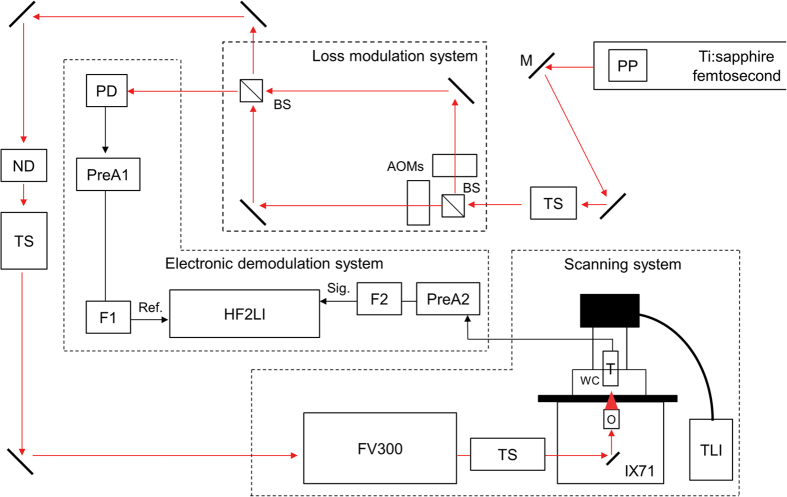
Experimental setup of 2PAM based on laser scanning. Dispersion pre-compensation was set within the laser to prevent the chirping effect caused by the AOMs in the loss modulation system. The red arrows in the figure represent laser light paths, while the black arrows indicate electrical signal connections. M, mirror; TS, telescope; BS, non-polarizing beam-splitter; AOM, acousto-optic modulator; PD, photo detector; ND, neutral density wheel; O, objective lens; WC, water cavity; T, ultrasonic transducer; PreA1 and PreA2, amplifiers (PR5678 and PR5660B, Olympus) for pre-amplification; F1 and F2, electronic low-pass filters (BLP-15+ and BLP-1.9+, Minicircuit); Ref., reference signal in electrical form; Sig., photoacoustic signals in electrical form; PP, dispersion pre-compensation prism pair; TLI, transmitted light illuminator for bright field observation.

After fully characterizing the spatial resolution of 2PAM, to raise the performance further, we increased the imaging rate by transforming the original mechanical-scanning 2PAM system into a laser-scanning one (Fig. 8; *see also* **Two-photon acoustic microscopy system setups** *in* **Methods**). A Chameleon Vision II (Coherent Inc.) Ti:sapphire laser with pre-compensation function providing ~16000 fs^2^ maximum negative dispersion was used as light source. The pulse width was ~140 fs. Also, a commercial galvo-mirror scanning inverted microscope (FV300 and IX-71, Olympus) was utilized for the following imaging applications. The immersion-type ultrasonic transducer (V303, Olympus) for photoacoustic signal detection was mounted on the sample stage. With an ultrasonic focal spot of around 36 mm^3^, the 3D optical scanning range remained well-within the ultrasonic focus, so that no mechanical translation of the transducer or sample during image acquisition was needed. Here, a HF2LI LIA (Zurich Instruments) was used in 2PAM for signal demodulation. This was because that HF2LI had a fast signal update rate which could keep up with the short pixel duration (~10 μs) of the galvo-mirror scanner.

The use of a femtosecond laser with a high repetition rate of 80 MHz enabled this 2PAM to sustain a high imaging rate same as a regular laser-scanning microscope with a large pixels number and a large FOV. For example, our simple system can achieve 0.38 fps for 512 × 512 pixels in a 350 × 350 μm^2^ FOV. Although an optical 2D scanning high-lateral-resolution PAM with a real-time *in vivo* imaging rate already exists, a FOV of only 50 × 50 μm^2^ with 60 × 60 pixels was demonstrated^8^. Furthermore, the lateral and axial resolutions of the system were 2.6 μm and ~30 μm, respectively. There was another literature reporting a high frame rate (~30 fps) *in-vivo* photoacoustic microscopy using 3.5 MHz ultrasonic transducer with a FOV of 250 × 250 μm^2^, yet the pixels number was only 100 × 100^7^. Also, the lateral and axial resolutions of the system were ~6 μm and ~500 μm, respectively. The low sampling rate and the poor spatial resolution was not enough for 3D high-resolution non-fluorescent imaging. As a consequence, we believe that this much improved frame rate, similar to that of the current real-time scanning fluorescence microscope, for such a wide FOV combined with sub-femtoliter spatial resolution will fulfill the high demand for future *in vivo* 3D imaging by using non-fluorescent dye/protein staining.

### *In vivo* and *ex vivo* 2PAM imaging applications

Here we demonstrate the first *ex vivo* and *in vivo* imaging applications by using the endogenous contrast provided by melanin with this laser scanning 2PAM (Fig. 8). The laser wavelength was 860 nm, and the optical power was less than 45 mW, in both *ex vivo* and *in vivo* imaging experiments. Laser safety was guaranteed by comparing the experimental conditions with a literature^23^, and no photo-damage had been observed during or after the imaging as well (*see* **Animal imaging experiments** *in* **Methods**).

**Figure 9:**
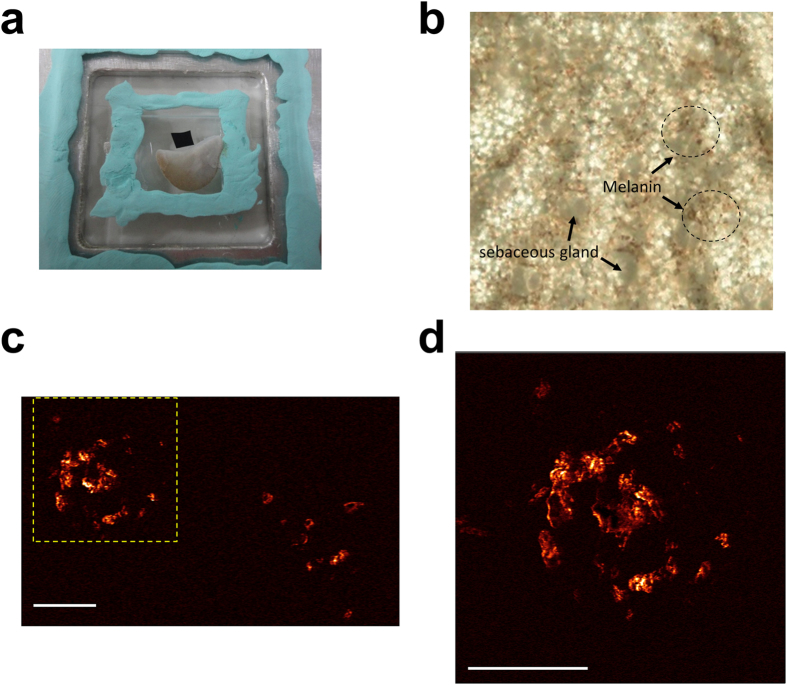
The *ex vivo* photoacoustic imaging of melanin distribution within a black mouse ear. (**a**) The photo of the ear tissue placed in a water cavity. The black tape above it was used for imaging system alignment. (**b**) The photo taken under bright field microscope, and we could see the melanin distribution and sebaceous gland (**c**) The two-photon acoustic image of the melanin distribution. The scale bar is 50 μm. (**d**) 3× zoomed-in image of the yellow dashed boxes in (**c**). The scale bar is 50 μm.

An ear tissue of a black mouse (C57BL/6J) was used in the *ex vivo* photoacoustic imaging for contrast identification. The melanin distribution within the skin surface was observed based on 2PA contrasts (Fig. 9). The identification of melanin was verified through the comparison with the image based on a bright-field microscope with the help of a pathologist. The laser modulation frequency, *f*, was 0.72 MHz. A NA0.8w objective lens (LUMPLFLN 40×, Olympus) was used to focus the modulated laser light on the ear tissue. Therefore, 2PA signal came from that at 2*f*, 1.44 MHz. The pixels number was 512 × 512 with pixel dwell time ~10 μs, so the acquisition time per frame was approximately 2.6 s.

**Figure 10:**
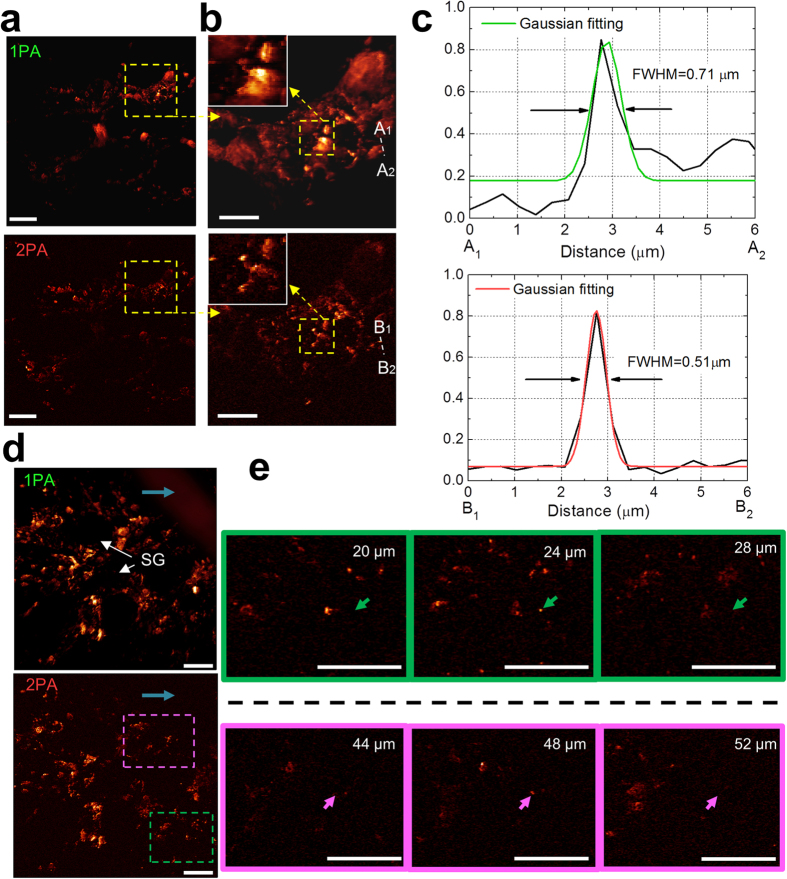
*In vivo* 1PA and 2PA images of melanin distribution near the basal layer of the epidermis^4^. (**a**) A 2D 1024 × 1024 pixels image at the depth ~20 μm. Dense mega-pixel number is for sufficiently high sampling frequency for lateral resolution verification. The scale bar is 50 μm. (**b**) Zoom-in images of the yellow dashed box in (**a**). The insets are the further enlarged details, and only 2PA image provides distinguished melanosomes. The scale bar is 20 μm. (**c**) The plotted response in (**b**) gives the upper bound for the lateral resolution. (**d**) A z projection of 512 × 512 pixels 3D images stack with 52 μm thickness and separation of 4 μm. SG (white arrow), sebaceous gland. The scale bar is 50 μm. In the 1PA case, an out-of-focus black fur of diameter ~60 μm (blue arrow) can be seen. However, this is not the case in 2PA case, where high optical sectioning capability is provided. (**e**) Zoom-in images of the green and purple dashed boxes of the 2PA case in (**d**) extended at different depths. Melanosomes (green and purple arrows) emerging in only a single image of a sequence is a proof that the axial resolution of 2PAM is <4 μm. The scale bar is 50 μm.

For the *in vivo* experiment, another black mouse (C57BL/6J) was anesthetized, and melanin distribution within its ear was imaged. In this *in vivo* imaging experiment, the laser modulation frequency, *f*, was 0.72 MHz, and a NA0.8w objective lens (LUMPLFLN 40×, Olympus) was used. Melanin distribution was observed based on both 1PA and 2PA contrasts. 1PA signal was from demodulation at *f*, 0.72 MHz, and 2PA signal was from that at 2*f*, 1.44 MHz. The attained lateral and axial resolutions of 2PAM were 0.51 μm and 2.41 μm, following the previous spatial resolution characterization respectively, as can be further verified from the images (Fig. 10). This *in vivo* imaging experiment proved the 2PAM as a non-fluorescent absorption-contrast *in vivo* microscopy with a 3D sub-femtoliter resolution 
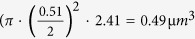
, with 0.8NA objective). Detailed discussions about the results of this *in vivo* imaging experiment are as follows.

## Discussion:

Images of 350 × 350 μm^2^ FOV were taken with high sampling rate, 1024 × 1024 pixels, and such dense pixels number were for the sub-micron lateral resolution verification of our imaging system (Fig. 10a). The images of melanin distribution at ~10 μm depth within the mouse ear from both 1PA and 2PA signals were taken simultaneously in an acquisition time of ~10 seconds. Blur and fin-like objects in 1PA image are some indistinguishable melanin clusters projected from depths out of focus because of the inferior spatial resolution. However, this is not the case for 2PA image, where only melanin on the focal plane is weighted the most, and more delicate details are provided. The black holes (white arrows) surrounded by melanin clusters are sebaceous glands (SG).

As we further zoomed into the images (Fig. 10b), we could see that only 2PAM distinguishes individual melanin particles (further enlarged in the inset) due to its superior lateral resolution compared to 1PA image, where the melanin particles are hidden in background. To carefully characterize the lateral resolution in both 1PA and 2PA images, a line pair, 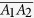
and 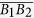, are dragged across an arbitrary region on the images. Then, intensity profiles of the line pair were analyzed.

From the selected line profiles plotted (Fig. 10c), we could see that 2PAM with NA0.8w objective could achieve 0.51 μm lateral resolution, measured as the FWHM of an intensity peak. This result is very close to the resolution limit of 2PAM, 0.48 μm, calculated from Eq. (1) (by substituting *λ*_*ex*_ = 0.86 and *NA* = 0.8 in to the equation). Meanwhile, 1PA image only has 0.71 μm lateral resolution, which is also close to the resolution limit of common PAM, 0.7 μm, calculated from equations for single photon absorption mechanism^9^. The lateral resolution improvement from 1PA to 2PA images, 0.71 μm to 0.51 μm, is a factor of 1.4. Such expected improvement was directly contributed from the 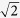
factor (in the denominator in Eq. (1)) of the two–photon nature. Without using very high NA objectives, we could still acquire sufficient resolution improvement by simply relying on the advantage from two-photon absorption mechanism. From the images (Fig. 10a–c), we directly verified the fine lateral resolution of 2PAM, and the results are consistent with the analysis carried out in the previous spatial resolution characterization. The verification of the axial resolution of 2PAM is also discussed in the following paragraphs.

A 52 μm thick 3D image stacks was collected with 4 μm depth step size (Fig. 10d). The stacks are piled from 350 × 350 μm^2^ images in a sampling rate of 512 × 512 pixels. An image pair of melanin distribution within the mouse ear from both 1PA and 2PA signals were taken simultaneously in an acquisition time of ~2.6 seconds. This 52 μm imaging depth should not be seen as an upper bound for 2PAM. This was because melanin was secreted from the basal layer, the bottom layer of epidermis of skin, and 52 μm was already the overall thickness of the epidermis layer of the mouse ear. The fact indicated that deeper penetration was still possible for 2PAM once contrasts existed at such depth. In this figure, 2PAM reveals its high optical sectioning capability by excluding an out-of–focus fur. This means that the photoacoustic images from 1PA signals were vulnerable to background signals, while 2PAM could eliminate this issue. To quantify the axial resolution of 2PAM, two regions on the 2PA images stack (purple and green dashed boxes) are extracted in a sequence at only three successive depths (Fig. 10e).

The axial resolution of 2PAM is further verified (Fig. 10e), as the observed melanosomes (green and purple arrows) emerged in only a single image of a depth sequence. From the capability of distinguishing information from different depth, we believed that 2PAM provided a fine optical sectioning with its <4 μm axial resolution. The result agrees with that calculated from Eq. (1) (by substituting *λ*_*ex*_ = 0.86 and *NA* = 0.8 in to the equation), 2.25 μm, and measured in the previous spatial resolution characterization section, 2.41 μm. Since our photoacoustic frequency of 2PA signal was of only 1.44 MHz in this *in vivo* imaging experiment, the fine micron-level axial resolution was provided from the localization of nonlinear excitation. This performance well lives up to our expectation of 2PAM discussed in the introduction. By taking the advantage of two-photon absorption mechanism, the predicament of common high-axial-resolution PAM operating at high frequency is solved. Also, echoing the introduction, due to the use of low frequency ultrasound, large acoustic FOV sustains *in vivo* optical scanning in large FOV and large pixels number for short image acquisition time in only several seconds.

Melanin is one of the major endogenous absorption sources of light within the UV to NIR spectrum with very weak fluorescence inside biological tissues^24^, and it plays a critical role in the diagnosis of various genetic abnormalities and pigmented skin diseases such as melanoma. Thus, *in vivo* melanin imaging is a particularly useful application of 2PAM as a demonstration of non-fluorescent absorption-based imaging. Since photoacoustic imaging modalities can image all endogenous or exogenous molecules at selected wavelengths, 2PAM is a potential solution to those biological and clinical applications where non-fluorescent staining dyes are preferred or non-fluorescent endogenous biomolecules are the selected imaging contrasts^25^. Moreover, due to the two-photon excitation nature, only excitation within a focal spot would generate 2PA signals. Intrinsic signal confinement from this nonlinear optical mechanism removes the necessity of temporal filtering as in common PAM.

In conclusion, microscopy based on non-fluorescent absorption dye staining is widely used in various fields of biomedicine for 400 years but lacks proper methodologies to realize its *in vivo* applications with a sub-femtoliter 3D resolution, unlike its fluorescent counterpart such as confocal fluorescence microscopy or two-photon fluorescence microscopy. Here by utilizing the non-radiative two-photon-absorption process, we have realized *in vivo* 2PAM with an optically determined sub-femtoliter 3D spatial resolution (0.49μm^3^) and a high frame rate (~2.6 s). This is achieved by femtosecond-laser excited two-photon acoustic contrasts, a loss modulation system for 2PA signals extraction, and a galvo-scanner for high imaging frame rates. This invention now allows the *in vivo* 3D microscopy with a sub-femtoliter resolution by using non-fluorescent staining dyes and should cover the territory of microscopy for high-resolution real-time *in vivo* imaging studies where fluorescent labeling probes are unavailable or not preferable.

## Methods

### Two-photon acoustic microscopy (2PAM) system setups

The microscope setup included four primary parts: a Ti:sapphire femtosecond laser, a loss modulation system, a scanning system, and an electronic demodulation system. The femtosecond Ti:sapphire laser was either Tsunami (Spectra Physics) or Chameleon Vision II (Coherent Inc.). The loss modulation system was a modification of the Mach-Zehnder interferometer with two acousto-optic modulators (AOM, R23080.5-3-.85-LTD and R23081-3-.85-LTD, Neos Technologies) on both arms. The beating effect from the frequency difference of the AOMs and the laser repetition rate led to pure sinusoidal intensity modulation of a frequency, *f*, on the laser light for photoacoustic excitation, and the nonlinear absorption within the focal excitation generated high harmonics of the modulation frequency, 2*f*, 3*f….* The scanning system included either mechanical scanning stages for resolution characterization or a commercial galvo-mirror scanning inverted microscope (FV300 and IX-71, Olympus) for the imaging applications. An immersion-type ultrasonic transducer (V303, Olympus) with a 1 MHz central frequency (ultrasonic focal spot was 2 × 2 × 9 mm^3^) for photoacoustic signal detection was mounted on the scanning stage, and the detected photoacoustic signals were transformed into electrical form for demodulation. The electronic demodulation system consisted of a lock-in amplifier (either SR844, Stanford Research, or HF2LI, Zurich Instruments) with an external reference fed from the loss modulation system for signal demodulation. By locking at the second harmonic of the modulation frequency, 2*f*, we were able to extract clean photoacoustic signals from the two-photon absorption.

### Step-edged method for axial resolution characterization

For the latter method, a sharp-edged object was imaged for the experimental quantification of the imaging system resolution. The edge-spread function (ESF) was estimated by measuring the edge response, and was fitted to an error function,


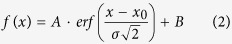


assuming that the beam profile was Gaussian. The line-spread function (LSF) was then calculated by differentiating the ESF. The resolution of the imaging system, defined as the FWHM (Full width half maximum) of the LSF, was thus derived.

### Animal imaging experiments

In the *ex vivo* photoacoustic imaging experiments, an ear tissue of a black mouse (C57BL/6J) was cut off and used for contrast identification. For the *in vivo* experiment, another black mouse (C57BL/6J) was anesthetized, ear-shaved, and placed in a water cavity mounted to the loading stage of the inverted microscope. Instead of water as our acoustic medium, we used malleable ultrasonic gel (Home Care Technology Co.) to prevent the mouse from drowning. After the *in vivo* imaging experiment on the mouse ear for around half an hour, the mouse was cleaned and placed on an electrical heating blanket, where it awoke spontaneously. All experimental animal procedures were carried out in conformity with the laboratory animal protocols approved by the Institutional Animal Care and Use Committee of National Taiwan University in Taiwan.

## Additional Information

**How to cite this article**: Lee, S.-Y. *et al. In vivo* sub-femtoliter resolution photoacoustic microscopy with higher frame rates. *Sci. Rep.* **5**, 15421; doi: 10.1038/srep15421 (2015).

## Figures and Tables

**Figure 1:**
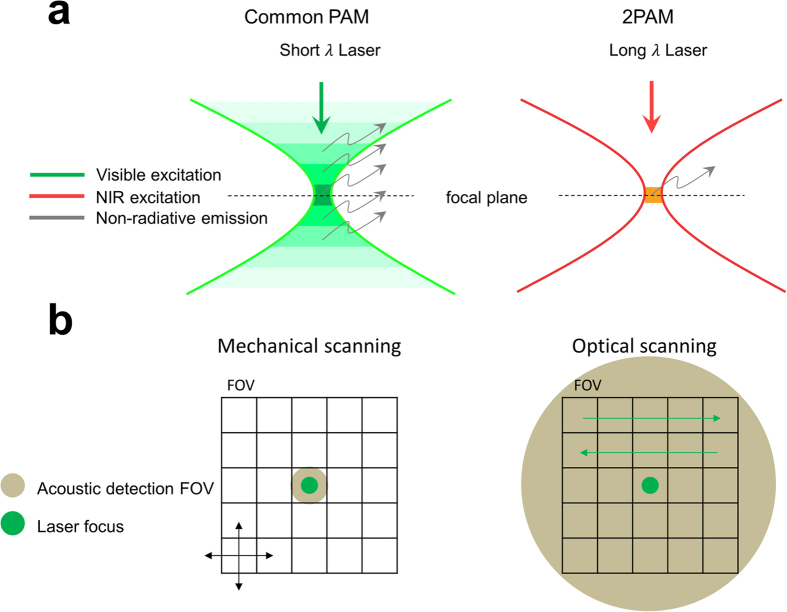
Comparisons between PAM and 2PAM. (**a**) In common PAM based on visible laser excitation process, temporal or spatial filtering is necessary to eliminate induced background and acquire the axial resolution. However, in 2PAM, with NIR excitation, only photons within the focal spot generate nonlinear photoacoustic signals. Therefore, intrinsic optical confinement determines the 3D spatial resolution. (**b**) Only mechanical scanning is applicable in current common PAM due to the small acoustic detection FOV. In contrast, 2PAM could be operated at low acoustic frequency creating large acoustic detection FOV. Optical scanning is thus possible.
